# Mismatch Repair Protein Expression in Endometrial Cancer: Assessing Concordance and Unveiling Pitfalls in Two Different Immunohistochemistry Assays

**DOI:** 10.3390/jpm13081260

**Published:** 2023-08-14

**Authors:** Hiroshi Yoshida, Waku Takigawa, Mayumi Kobayashi-Kato, Tadaaki Nishikawa, Kouya Shiraishi, Mitsuya Ishikawa

**Affiliations:** 1 Department of Diagnostic Pathology, National Cancer Center Hospital, 5-1-1, Tsukiji, Chuo-ku, Tokyo 104-0045, Japan; 2 Department of Gynecology, National Cancer Center Hospital, 5-1-1, Tsukiji, Chuo-ku, Tokyo 104-0045, Japan; 3 Department of Medical Oncology, National Cancer Center Hospital, 5-1-1, Tsukiji, Chuo-ku, Tokyo 104-0045, Japan; tnishika@ncc.go.jp; 4 Division of Genome Biology, National Cancer Center Research Institute, 5-1-1, Tsukiji, Chuo-ku, Tokyo 104-0045, Japan

**Keywords:** mismatch repair protein, immunohistochemistry, endometrial cancer, concordance, artifact

## Abstract

This study aimed to compare the concordance and interchangeability of the Dako/Agilent and Ventana/Roche mismatch repair (MMR) immunohistochemistry (IHC) assays commonly used in pathology. It also aimed to provide diagnostic insights by examining the frequency and characteristics of the dot-like artifact observed in MLH1 M1 clone staining in endometrial cancer. Fifty endometrial cancer cases with MMR deficiency, excised between 2011 and 2018, were included in the study. IHC was performed using primary antibody clones from Ventana/Roche (MLH1, clone M1; MSH2, G219-1129; MSH6, SP93; PMS2, A16-4) and Dako/Agilent (MLH1, ES05; MSH2, FE11; MSH6, EP49; PMS2, EP51). Both assays were conducted using respective autostainers. The Dako/Agilent assay showed a loss of MLH1 in 26 cases, MSH2 in 12 cases, MSH6 in 23 cases, and PMS2 in 28 cases. The two assays had a complete agreement in MMR protein expression or loss. The dot-like artifact in MLH1 M1 clone staining was observed in 77% (20/26) of cases, predominantly in the surface area of the tumor, ranging from 5% to 40% (median: 10%). These findings highlight the high concordance between the MMR-IHC assays and emphasize the importance of considering the dot-like artifact in MLH1 M1 clone staining when diagnosing endometrial cancer with MMR deficiency.

## 1. Introduction

Endometrial cancer is the second most prevalent gynecologic cancer in both resource-abundant and resource-limited countries [1]. Recent advancements in genomic analysis have uncovered that approximately 20–30% of endometrial cancer cases exhibit a mismatch repair deficiency (dMMR) phenotype [2,3,4]. dMMR is caused by genetic or epigenetic alterations of any of the mismatch repair genes (*MLH1, MSH2, MSH6, PMS2*, or *EPCAM*) [5,6]. Immunohistochemistry (IHC) of MMR proteins has emerged as a widely employed method for detecting the dMMR phenotype in endometrial cancer. The MMR IHC assay now serves at least three crucial clinical roles in endometrial cancer [3,7,8,9,10]. It serves as a molecular classification [3,8,10,11], a companion diagnostic for immunotherapy [7,10,12], and a secondary screening for Lynch syndrome [9,10]. Regarding molecular classification, various guidelines, and the recently published FIGO2023 staging encourage its implementation for all endometrial cancers, explicitly highlighting the requirement for dMMR assessment through IHC [8,10,11]. In immunotherapy applicability, MMR IHC is preferred over PCR-based microsatellite instability testing for endometrial cancer [7]. Universal screening for Lynch syndrome through MMR IHC in all endometrial cancer cases is also suggested by certain guidelines [9,10,11]. Under such circumstances, the significance of the MMR-IHC assay in patients with endometrial cancer has become increasingly evident. 

However, clonal differences in primary antibodies have been reported to cause variations in staining characteristics in MMR IHC [13,14,15,16,17]. These variations can potentially lead to misleading results in MMR IHC in endometrial cancer. Moreover, the concordance rates of MMR IHC results between Dako/Agilent and Ventana/Roche diagnostics, which are currently gaining popularity [13,17,18], are not well-established in the context of endometrial cancer. Additionally, the most prevalent cause of dMMR in endometrial cancer, *MLH1* promoter methylation [19,20], results in the concurrent loss of MLH1 and PMS2 staining patterns in IHC [19,21,22]. However, a previous study primarily focusing on colorectal cancer patients documented the presence of “dot-like artifacts” in MLH1 staining [17,23], which can erroneously categorize dMMR cases as MMR proficient (pMMR). While similar artifacts have been reported in endometrial cancer [22,24], their frequency and characteristics remain inadequately elucidated. 

This study aims to compare the results of MMR IHC assays by Dako/Agilent and Ventana/Roche, extensively utilized in current pathological practice, to investigate their concordance rates and determine the interchangeability of IHC results. Additionally, we seek to clarify the frequency and characteristics of the dot-like artifact associated with the MLH1 M1 clone in endometrial cancer, guiding us to avoid diagnostic pitfalls.

## 2. Materials and Methods 

### 2.1. Case Selection 

Patients with histologically confirmed stage I to III (FIGO 2008) endometrial cancer diagnosed between 2011 and 2018 were identified from our institution’s tumor registry database. In a previous study on the molecular classification of endometrial cancer [25], we identified four molecular subtypes (*POLE*mut, dMMR, p53abn, and NSMP) in 265 endometrial cancer cases using *POLE* sequencing and immunohistochemistry. Among these, we selected 50 cases of dMMR endometrial cancer based on IHC using the Dako/Agilent assay. All cases were independently reviewed by at least two gynecological pathologists, and the pathological diagnoses were confirmed according to the 2020 World Health Organization tumor classification [4]. Clinicopathological data were retrospectively collected for each patient. The study was approved by the Institutional Review Board of the National Cancer Center in Japan (#2017-331) and conducted in accordance with the Declaration of Helsinki. Informed consent was waived due to the retrospective nature of the study. 

### 2.2. IHC Analysis and Interpretation

Surgically resected specimens were fixed in 10% neutral-buffered formalin for 24–72 h and embedded in paraffin. One representative whole 4 μm thick section was analyzed using immunohistochemistry (IHC). Detailed information on the antibodies used for immunohistochemical staining of MMR proteins is provided in Table 1. Dako/Agilent antibodies, including MLH1 (clone ES05), MSH2 (clone FE11), MSH6 (clone EP49), and PMS2 (clone EP51), were stained using Dako’s automated staining system (LINK48, Dako, CA, USA). Ventana/Roche antibodies, including MLH1 (clone M1), MSH2 (clone G219-1129), MSH6 (clone SP93), and PMS2 (clone A16-4), were stained using Ventana’s automated staining system (Benchmark XT). All staining procedures were performed according to the manufacturer’s recommendations.

The expression of MMR proteins was assessed by pathologists following established pathological guidance [7,21,22,26]. Specifically, after confirming appropriate staining of the nuclei in internal positive controls within the tissue sections, the expression of MMR proteins in the nuclei of tumor cells was evaluated. The assessment included evaluating for retained or lost expression for each of MLH1, MSH2, MSH6, and PMS2 proteins. Adjacent normal epithelial cells, stromal cells, and inflammatory cells with intact nuclear staining served as internal positive controls. It has been reported that MMR protein loss can occur in only a subset of tumor cells, known as “subclonal loss.” In this study, we aimed to investigate whether staining characteristics remain consistent even in cases of subclonal loss in endometrial cancer. Therefore, data on the subclonal loss of MMR proteins were collected. Subclonal loss was defined as areas with retained and lost nuclear staining, with intervening stromal cell positivity as an obligatory internal positive control [26,27].

Furthermore, the MLH1 M1 clone has been reported to exhibit dot-like artifacts [22,23]. Dot-like artifacts are described as “granular, dot-like staining with MLH1-IHC in the nuclei of the tumor cells was distinct from the homogenous staining in the internal control” [23]. Information on the percentage of tumor area with artifacts and the location within the tumor where artifacts were observed was recorded in relation to dot-like artifacts. Artifacts covering less than 5% of the area were excluded as they were unlikely to be clinically significant. 

### 2.3. Statistical Analysis

All descriptive statistics were calculated, and Fisher’s exact test was performed with JMP 10.0.0 software (SAS Institute Inc., Cary, NC, USA). A *p*-value < 0.05 was considered to be statistically significant.

## 3. Results

**Figure 1:**
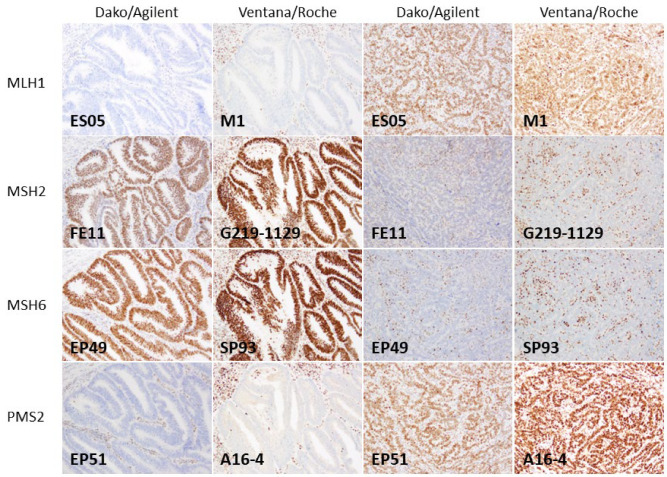
Immunohistochemistry for MMR proteins using two different assays. The figures display the immunohistochemical staining results obtained from the Dako/Agilent and Ventana/Roche assays, illustrating cases with protein loss and retained protein expression. The top row corresponds to MLH1, the second to MLH2, the third to MSH6, and the final to PMS2. All original images are captured at a magnification of 200×.

A total of 50 cases of dMMR endometrial cancer, excised between 2011 and 2018, were included in this study. The patients had a median age of 56 (28 to 77 years). All cases were surgical specimens, and no biopsy materials were included. The histological types consisted of 22 cases of endometrioid carcinoma (G1), 15 cases of endometrioid carcinoma (G2), 4 cases of endometrioid carcinoma (G3), 4 cases of mixed carcinoma (serous carcinoma and endometrioid carcinoma), 3 cases of serous carcinoma, 1 case of dedifferentiated carcinoma, and 1 case of carcinosarcoma. Among the specimens, 64% (32/50) utilized FFPE blocks with a storage period of up to 10 years for immunostaining. The results of MMR immunohistochemical staining using Dako/Agilent’s clones showed loss of MLH1 in 26 cases, loss of MSH2 in 12 cases, loss of MSH6 in 23 cases, and loss of PMS2 in 28 cases. For these cases, immunohistochemical staining was performed using primary antibody clones from Ventana/Roche (MLH1, clone M1; MSH2, clone G219-1129; MSH6, clone SP93; PMS2, clone A16-4), and the results were compared with those of immunohistochemical staining from the Dako/Agilent assay (Table 2). The protein expression or loss results were 100% concordant for all MMR proteins. Representative images of immunohistochemical staining are shown in Figure 1.

**Figure 2:**
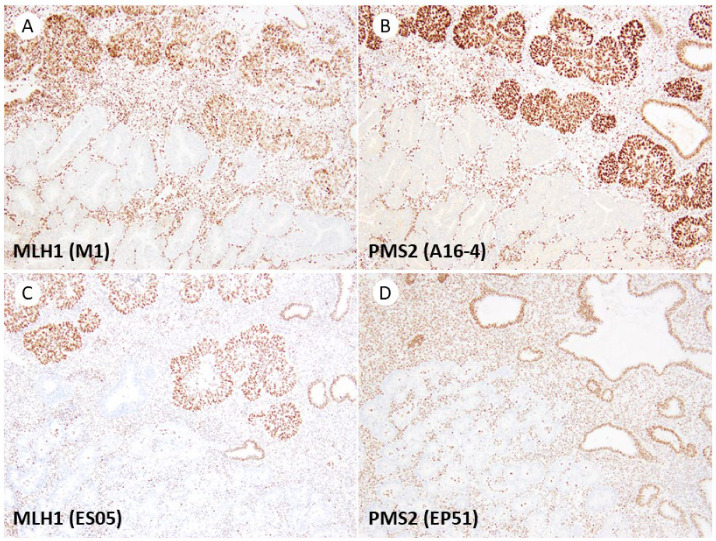
Subclonal loss of MLH1 and PMS2. (**A**) MLH1 (M1 clone). Tumor glands exhibiting protein expression are visible in the upper half of the image, while tumor glands with protein loss can be observed in the lower half. (**B**) MLH1 (M1 clone) and PMS2 (A16-4) demonstrate similar patterns of protein loss. (**C**,**D**) Comparable staining patterns are also detected in the Dako/Agilent assay, with MLH1 (ES05) and PMS2 (EP51). All original images are captured at a magnification of 200×.

Cases showing subclonal loss of the MMR protein were identified in 5 out of 50 cases, with focal loss of MLH1 and PMS2 observed. The staining results for subclonal loss were consistent between the Dako/Agilent assay and Ventana/Roche assay. Representative images are shown in Figure 2.

**Figure 3:**
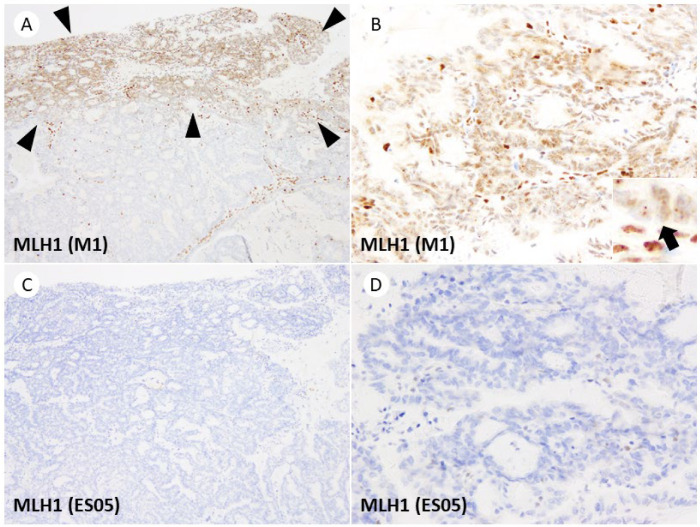
Dot-like artifacts of MLH1 (M1 clone). (**A**) Weak staining in the superficial area of the tumor is visible at low magnification (arrowheads, 40×). (**B**) Higher magnification (100×) reveals faint dot-like staining in the nuclei of tumor cells, distinctly different from the nuclei of internal positive control cells in the stroma (inset, arrow, lower half represents the nucleus of internal positive control cells, 400×). (**C**,**D**) Dako/Agilent MLH1 (ES05) displays no dot-like staining in the same area of the same case ((**C**), 40×; (**D**), 200×).

The dot-like artifact of the MLH1 M1 clone, previously reported in the literature, was observed in 77% (20/26) of cases. The area of the tumor showing the dot-like artifact ranged from 5% to 40% (median: 10%), and in 90% (18/20) of cases, it was observed on the surface area of the tumor near the uterine cavity. No significant correlation was found between specific histological types or subclonal loss and the frequency of artifact occurrence. Representative images of the dot-like artifact are shown in Figure 3.

**Figure 4:**
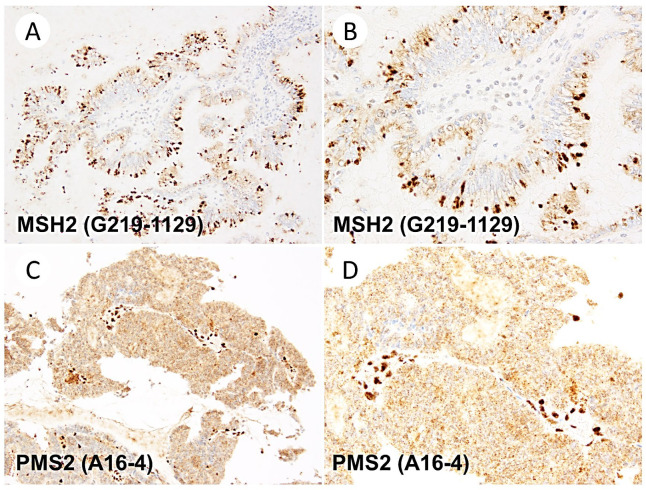
Rare artifacts identified in the present study, apart from the dot-like artifacts associated with the M1 clone. (**A**,**B**) Cytoplasmic granular staining of MSH2. (**C**,**D**) Very fine punctate cytoplasmic staining of PMS2. Original images are captured at a magnification of 200× and 400× for (**A**,**C**) and (**B**,**D**), respectively.

In addition, rare artifacts were observed during the MMR IHC assessment. These included the cytoplasmic positivity of the MSH2 clone (3/12, 25%), the cytoplasmic positivity of the PMS2 clone (3/28, 11%), and luminal staining of the MLH1 clone M1 (1/26, 3.8%). These artifacts were limited to areas comprising less than 10% of the tumor tissue. Representative images of these artifacts are shown in Figure 4.

## 4. Discussion

This study compared the results of two different MMR immunohistochemical assays using surgical specimens from 50 cases of dMMR endometrial cancer. The study showed a high level of agreement between the two assays, indicating their compatibility. However, it also highlighted the presence of artifacts, particularly in MLH1 (M1 clone) staining, which could lead to misinterpretation of results. These artifacts were frequently observed on the surface side of the tumor in most cases.

Regarding immunostaining for dMMR in endometrial cancer, the Dako/Agilent assay and the Ventana/Roche assay showed complete concordance (100%; 50/50) in the present study. Not only were the results of protein loss matched, but the subclones of the tumor that exhibited protein loss were also matched in cases of subclonal loss. Several external quality assurance (EQA) schemes have published results for MMR IHC runs [13,14,15,16,17,18]; however, there is a lack of data evaluating the concordance rates of MMR IHC using two different assays for the identical endometrial cancer specimens within the same laboratory. In the NordiQC’s EQA runs, tissue microarrays consisting of one pMMR and two dMMR colorectal cancers, in addition to control tissue, were distributed to each participating institution for MMR-IHC evaluation. In the MLH1 EQA run, 75% (36/48) of the ES05 clone (concentrated antibody) and 70% (70/100) of the M1 clone (ready-to-use antibody) yielded sufficient results [17]. The MSH2 EQA run indicated a sufficient result rate of 62% (18/26) for the FE11 clone (concentrated antibody), whereas the G219-1129 clone (ready-to-use antibody) achieved an 86% (94/110) success rate [14]. Regarding the MSH6 EQA, the EP49 clone (concentrated antibody) demonstrated a 96% (46/48) sufficient result, while the SP93 clone (ready-to-use antibody) achieved a 97% (60/62) success rate [15]. For the PMS2 EQA run, the EP51 clone (concentrated antibody) exhibited a 93% (37/40) sufficient result rate, while the A16-4 clone (ready-to-use antibody) showed a 49% (16/33) rate of satisfactory results [16]. Based on these results, the NordiQC EQA-run reports concluded that the MLH1, PMS2, MSH6, and MSH2 antibody clones used in this study consistently produced optimal staining results [14,15,16,17]. The UKNQAS EQA runs of MMR-IHC also employed colon tumor tissue for immunostaining. According to a report summarizing the UKNQAS EQA runs of MMR-IHC (2011–2019, N = 4447), all MMR-IHC antibody clones used in our study received an average assessment score of 14 or higher (four raters assigned a score of 1–5 to each stained slide, with a sum of scores of 13 or higher considered “Acceptable”) [13]. While these EQA results anticipated good agreement between the Ventana/Roche and Dako/Agilent MMR-IHC assays, scarce data comparing the two assays in endometrial cancer existed. The present study contributes to establishing the compatibility of results obtained from the two assays in endometrial cancer.

However, as detailed in the following section, it is essential to note that the MLH1 clone M1 exhibits a high frequency of dot-like artifacts. These artifacts can lead to misclassification and lower the agreement rate between the assays. Given the current state of molecular classification in endometrial cancer [8,9,11] and the growing adoption of universal screening for Lynch syndrome [9,28,29], it is assumed that the MMR IHC will be performed on the vast majority of endometrial cancers. Although other vendors offer different MMR IHC assays [13,14,15,16,17], the strong concordance observed between these two widely used assays provides valuable information when considering compatibility with other assays.

Dot-like artifacts were frequently observed in the M1 clone of MLH1, and it is well-documented that dot-like artifacts in M1 clones can lead to the misclassification of dMMR cases as pMMR [22,23]. In 2017, Markow et al. highlighted the nucleolar staining pattern of MLH1 IHC as a potential pitfall in MMR-IHC assessment [30]. Subsequently, Niu et al. described heterogeneous punctate nuclear staining in MLH1 IHC (M1 clone) in six endometrial cancer cases previously classified as isolated PMS2-loss [24]. Importantly, when MLH1-IHC was repeated using the ES05 clone, all cases showed complete negativity, and the *MLH1* promoter was found to be methylated [24]. Similarly, Dasgupta et al. reported granular, dot-like nuclear staining of MLH1 (M1 clone) in three cases of colorectal cancer [23]. They noted that this artifact was detected in 6.3% (2/32) of colorectal cancers but was absent in endometrial cancers (0/4) [23]. In our study, dot-like artifacts were identified in 77% (20/26) of cases, indicating a high frequency of these artifacts in endometrial cancer.

The etiology of the dot-like artifact observed with the M1 clone remains fully understood, and the factors influencing its frequency and extent of occurrence are unclear. Loughrey et al. reported this artifact in 9 out of 16 PMS2-lost colorectal cancer cases, with 4 of them showing somatic *BRAF* V600E variants [31]. They hypothesized that the punctate nuclear staining pattern indicates a non-functional MLH1 protein variant that retains antigenicity, possibly resulting from the somatic mutation [31]. However, the same explanation may not apply to endometrial cancer as to colorectal cancer, as there is no correlation between the *MLH1* promoter methylation and the *BRAF* V600E variant in endometrial cancer. Notably, the NordiQC EQA report on MLH1 observed a dot-like artifact in colon adenocarcinoma tissue when immunostained with the M1 clone. The report suggests that excessively long incubation times of tyramide-based amplification reagents may contribute to the occurrence of this artifact [17].

In our study, the observation that the dot-like artifact predominantly appears in the superficial area of the tumor suggests a potential association between the artifact and tissue fixation status or cold ischemic time. Furthermore, in endometrial biopsy specimens, which primarily sample the superficial layer of the tumor, the dot-like artifact might be more frequently observed and cover a larger area of the tumor. However, this hypothesis needs to be confirmed in future studies. We should recognize the varying frequencies of artifacts associated with different antibody clones and their preferred sites to avoid misinterpretation. Careful comparison of nuclear staining in tumor cells with internal positive control cells at high magnification is recommended, as hasty judgments based on low-magnification observations alone should be avoided. Since the range of artifact occurrence was 5–40% (median 10%), affecting only a small portion of the tumor in most cases, by diligently examining the entire tumor, determination of dMMR status can be achieved.

This study also identified several abnormal staining patterns, although they were less frequent than the dot-like staining observed with MLH1 (M1 clone). Cytoplasmic staining of MSH2 and PMS2 has previously been reported in the EQA run reports of NordiQC using colon adenocarcinoma tissue [14,16]. These artifacts were present in less than 10% of the tumors. When carefully compared to the staining of internal positive controls, they are not expected to pose a problem for determining dMMR status. The British Association of Gynecological Pathologists provides guidance suggesting that in cases where atypical staining patterns are challenging to interpret, the case should be re-stained at a different tumor site or using a biopsy specimen. In some uninterpretable cases, a comprehensive judgment should be made with the MSI test results [22].

As the significance of MMR IHC in endometrial cancer continues to grow, understanding the concordance rates of commonly used antibodies and the characteristics of artifacts that could potentially lead to misinterpretation, as demonstrated in this study, is crucial for ensuring patient safety and avoiding harm. The determination of dMMR is essential for the recent molecular classification of endometrial cancer and is encouraged in the latest 2023 FIGO staging system [8]. Our approach to endometrial carcinoma has undergone significant transformation due to the groundbreaking research conducted by the Cancer Genome Atlas (TCGA) Research Network. For decades, risk stratification of endometrial cancer relied on histopathological characteristics, such as tumor grade, histotype, depth of myometrial invasion, and cervical stromal invasion. However, a pivotal turning point occurred in 2013 when TCGA performed an integrated molecular characterization of endometrial cancer, identifying four prognostically relevant groups based on mutational burden and somatic copy-number variations [2]. Subsequent research revealed that more straightforward and cost-effective immunohistochemical and molecular tests could be surrogates for the intricate and expensive TCGA analyses [32,33,34,35]. These surrogate tests efficiently identified four distinct molecular prognostic groups: *POLE*-mutated, dMMR, p53-abnormal, and “no specific molecular profile” (NSMP) [4]. This approach involves an initial search for the *POLE* exonuclease domain mutation, followed by the determination of dMMR using immunohistochemistry (IHC), and ultimately the determination of p53 abnormalities using IHC. These findings have significantly impacted the European (ESGO-ESTRO-ESP) guidelines for managing endometrial cancer, prompting their recent update to incorporate these molecular prognostic groups [10]. Including these groups in the guidelines ensures a more personalized and tailored approach to managing endometrial cancer, fostering improved patient outcomes, and advancing the field of gynecological oncology. Thus, failure to identify dMMR cases may result in the misclassification of tumors as p53abn-type or NSMP-type, misclassifying “multiple classifier” cases with both dMMR and *TP53* alterations as p53abn-type [8]. This misclassification may lead to unnecessary and excessive postoperative treatments for patients [11].

In addition, failure in Lynch syndrome screening may result in inadequate subsequent patient care, such as colorectal cancer screening, and the provision of insufficient information to patients and their families. Universal screening for Lynch syndrome in endometrial cancer has emerged as a crucial strategy to identify at-risk individuals and facilitate timely interventions. Lynch syndrome, a hereditary cancer predisposition syndrome, increases the lifetime risk of developing various cancers, particularly colorectal and endometrial cancers. The screening involves testing for microsatellite instability or deficient mismatch repair using immunohistochemistry in endometrial cancer tissue. Due to the relatively high prevalence of Lynch syndrome in endometrial cancer patients, universal screening has gained traction as an effective means to detect cases that might otherwise be missed. Identifying Lynch syndrome early allows for appropriate genetic counseling, surveillance, and preventive measures for the patient and their at-risk relatives. Several professional organizations, such as the National Comprehensive Cancer Network (NCCN) and the Society of Gynecologic Oncology (SGO), now recommend universal screening for Lynch syndrome in all newly diagnosed endometrial cancer cases [36].

Moreover, in the context of advanced or recurrent cancer, the failure to determine the dMMR status may result in missed opportunities for immunotherapy. Anti-PD-1/PD-L1 immunotherapy demonstrates broad efficacy against various cancers [37]. Biomarkers aiding the prediction of anti-PD-1 immunotherapy responsiveness have been identified. These biomarkers involve the PD-1 receptor and ligand expression analysis [38], high tumor mutational burden [39], and MMR deficiency [40,41]. MMR deficiency-associated cancers display these biomarkers due to uncorrected mutations from deficient MMR proteins, resulting in accumulated mutations. DNA mutations prompt novel epitope (neoantigen) expression, inciting immune responses and PD-1 ligand upregulation [42]. In anti-PD-1 immunotherapy, the antibody obstructs PD-1 receptor binding on T-cells’ surface, preventing apoptosis upon tumor interaction. Remarkably, this therapy targets biomarkers rather than tissue types. Though MMR deficiency triggers malignancies in various tissues, endometrial cancer is the most commonly tested gynecological cancer for dMMR [40]. Initial trials involving MMR-deficient gynecologic cancers showed responsiveness to anti-PD-1 immunotherapy [12,43]. The most extensive study included 90 patients (79 patients available for analysis) with endometrial cancer. The cohort exhibited a 48% objective response rate. The median progression-free and overall survival were 13.1 months and not reached [44]. Although complete responses were infrequent, notable partial response durations underscore the potential of immune checkpoint inhibitors as adjunctive MMR-deficient endometrial cancer treatment. Overall, MMR-IHC is a valuable tool in precision medicine, ensuring appropriate treatment selection and ultimately improving outcomes for patients with endometrial cancer who are candidates for immune checkpoint inhibitor therapy.

Furthermore, the compatibility of results obtained from the Dako/Agilent MMR-IHC assay with the Ventana/Roche assay provides valuable information for future analyses integrating data from cases evaluated using different MMR-IHC assays.

There are several limitations to be considered in this study. Although we selected two representative clones considering their increasing use in the market, we did not provide compatibility data for other less frequently used clones. Other factors, such as the detection system and automated staining equipment, may also influence staining results [13,14,15,16,17]. The possibility that staining may be affected by factors other than antibody clonal variation should not be overlooked. Additionally, the discussion on the causes of artifacts in the M1 clone still needs to be fully explored, and this aspect should be addressed in future investigations.

In conclusion, this study demonstrates good agreement between two commonly used MMR IHC assays for endometrial cancer. It also highlights the frequent dot-like artifacts in MLH1 (M1 clone) staining. These findings have practical implications for the application of MMR IHC in endometrial cancer and underscore the need for careful interpretation of staining results to avoid misclassification.

## Figures and Tables

**Table 1:** Primary antibodies used for immunohistochemistry of MMR proteins.

Antibody	Clone	Manufacturer	Dilution	Antigen Retrieval	Incubation	Detection System	Auto Stainer
MLH1	ES05	Dako/Agilent	1:200	Dako PT Link *	60 min RT	Envision	Link48
	M1	Ventana/Roche	Prediluted	CC1 64 min	24 min 37 °C	OptiView	Benchmark XT
MSH2	FE11	Dako/Agilent	1:200	Dako PT Link *	60 min RT	Envision + linker	Link48
	G219-1129	Ventana/Roche	Prediluted	CC1 40 min	12 min 37 °C	OptiView	Benchmark XT
MSH6	EP49	Dako/Agilent	1:400	Dako PT Link *	60 min RT	Envision	Link48
	SP93	Ventana/Roche	Prediluted	CC1 64 min	12 min 37 °C	OptiView	Benchmark XT
PMS2	EP51	Dako/Agilent	1:200	Dako PT Link *	60 min RT	Envision	Link48
	A16-4	Ventana/Roche	Prediluted	CC1 92 min	44 min 37 °C	OptiView + Amp	Benchmark XT

* Dako PT Link high-pH, 97 °C 20 min.

**Table 2:** Concordance of immunohistochemical staining results of MMR proteins between two different assays.

MLH1		ES05 (Dako/Agilent)	
		Retained	Lost
M1 (Ventana/Roche)	Retained	24	0
	Lost	0	26
MSH2		FE11 (Dako/Agilent)	
		Retained	Lost
G219-1129 (Ventana/Roche)	Retained	38	0
	Lost	0	12
MSH6		EP49 (Dako/Agilent)	
		Retained	Lost
SP93 (Ventana/Roche)	Retained	27	0
	Lost	0	23
PMS2		EP51 (Dako/Agilent)	
		Retained	Lost
A16-4 (Ventana/Roche)	Retained	22	0
	Lost	0	28

## Data Availability

The datasets generated and analyzed during the current study are available from the corresponding author upon reasonable request.

## References

[1] Sung H., Ferlay J., Siegel R.L., Laversanne M., Soerjomataram I., Jemal A., Bray F. (2021). Global Cancer Statistics 2020: GLOBOCAN Estimates of Incidence and Mortality Worldwide for 36 Cancers in 185 Countries. CA Cancer J. Clin..

[2] Levine D.A., Getz G., Gabriel S.B., Cibulskis K., Lander E., Sivachenko A., Sougnez C., Lawrence M., Kandoth C., Dooling D. (2013). Integrated genomic characterization of endometrial carcinoma. Nature.

[3] Lu K.H., Broaddus R.R. (2020). Endometrial Cancer. N. Engl. J. Med..

[4] WHO Classification of Tumours Editorial Board (2020). Female Genital Tumours, WHO Classification of Tumours.

[5] Cerretelli G., Ager A., Arends M.J., Frayling I.M. (2020). Molecular pathology of Lynch syndrome. J. Pathol..

[6] Deshpande M., Romanski P.A., Rosenwaks Z., Gerhardt J. (2020). Gynecological Cancers Caused by Deficient Mismatch Repair and Microsatellite Instability. Cancers.

[7] Bartley A.N., Mills A.M., Konnick E., Overman M., Ventura C.B., Souter L., Colasacco C., Stadler Z.K., Kerr S., Howitt B.E. (2022). Mismatch Repair and Microsatellite Instability Testing for Immune Checkpoint Inhibitor Therapy: Guideline from the College of American Pathologists in Collaboration With the Association for Molecular Pathology and Fight Colorectal Cancer. Arch. Pathol. Lab. Med..

[8] Berek J.S., Matias-Guiu X., Creutzberg C., Fotopoulou C., Gaffney D., Kehoe S., Lindemann K., Mutch D., Concin N., Endometrial Cancer Staging Subcommittee, FIGO Women’s Cancer Committee (2023). FIGO staging of endometrial cancer: 2023. Int. J. Gynaecol. Obstet..

[9] (2023). NCCN Guidelines Version 2.2023 Uterine Neoplasm. https://www.nccn.org/professionals/physician_gls/pdf/uterine.pdf.

[10] Concin N., Creutzberg C.L., Vergote I., Cibula D., Mirza M.R., Marnitz S., Ledermann J.A., Bosse T., Chargari C., Fagotti A. (2021). ESGO/ESTRO/ESP Guidelines for the management of patients with endometrial carcinoma. Virchows Arch..

[11] Oaknin A., Bosse T.J., Creutzberg C.L., Giornelli G., Harter P., Joly F., Lorusso D., Marth C., Makker V., Mirza M.R. (2022). Endometrial cancer: ESMO Clinical Practice Guideline for diagnosis, treatment and follow-up. Ann. Oncol..

[12] Marabelle A., Le D.T., Ascierto P.A., Di Giacomo A.M., De Jesus-Acosta A., Delord J.P., Geva R., Gottfried M., Penel N., Hansen A.R. (2020). Efficacy of Pembrolizumab in Patients with Noncolorectal High Microsatellite Instability/Mismatch Repair-Deficient Cancer: Results From the Phase II KEYNOTE-158 Study. J. Clin. Oncol..

[13] Parry S., Dodson A. Mismatch Repair Protein antibodies and their performance in the UK National External Quality Assessment Scheme for Immunocytochemistry and In-situ Hybridisation. Proceedings of the 31st European Congress of Pathology.

[14] (2019). MSH2 Run 57, Nordic Immunohistochemical Quality Control. https://www.nordiqc.org/downloads/assessments/122_82.pdf.

[15] (2021). MSH6 Run 61, Nordic Immunohistochemical Quality Control. https://www.nordiqc.org/downloads/assessments/145_83.pdf.

[16] (2021). PMS2 Run 62, Nordic Immunohistochemical Quality Control. https://www.nordiqc.org/downloads/assessments/146_84.pdf.

[17] (2023). MLH1 Run 67, Nordic Immunohistochemical Quality Control. https://www.nordiqc.org/downloads/assessments/171_81.pdf.

[18] (2020). Summary Report–Run 118 MMR (MLH1, PMS2, MSH2, MSH6).

[19] Hampel H., Pearlman R., de la Chapelle A., Pritchard C.C., Zhao W., Jones D., Yilmaz A., Chen W., Frankel W.L., Suarez A.A. (2021). Double somatic mismatch repair gene pathogenic variants as common as Lynch syndrome among endometrial cancer patients. Gynecol. Oncol..

[20] Simpkins S.B., Bocker T., Swisher E.M., Mutch D.G., Gersell D.J., Kovatich A.J., Palazzo J.P., Fishel R., Goodfellow P.J. (1999). MLH1 promoter methylation and gene silencing is the primary cause of microsatellite instability in sporadic endometrial cancers. Hum. Mol. Genet..

[21] Casey L., Singh N. (2021). POLE, MMR, and MSI Testing in Endometrial Cancer: Proceedings of the ISGyP Companion Society Session at the USCAP 2020 Annual Meeting. Int. J. Gynecol. Pathol..

[22] Naveena Singh R.W., Tchrakian N., Allen S.-G., Clarke B., Gilks C.B. (2020). Interpretation and Reporting Terminology for Mismatch Repair Protein Immunohistochemistry in Endometrial Cancer, BAGP Guidance Document: MMR Immunohistochemistry Interpretation and Terminology.

[23] Dasgupta S., Ewing-Graham P.C., Groenendijk F.H., Stam O., Biermann K.E., Doukas M., Dubbink H.J., van Velthuysen M.F., Dinjens W.N.M., Van Bockstal M.R. (2020). Granular dot-like staining with MLH1 immunohistochemistry is a clone-dependent artefact. Pathol. Res. Pract..

[24] Niu B.T., Hammond R.F.L., Leen S.L.S., Faruqi A.Z., Trevisan G., Gilks C.B., Singh N. (2018). Artefactual punctate MLH1 staining can lead to erroneous reporting of isolated PMS2 loss. Histopathology.

[25] Asami Y., Kobayashi Kato M., Hiranuma K., Matsuda M., Shimada Y., Ishikawa M., Koyama T., Komatsu M., Hamamoto R., Nagashima M. (2023). Utility of molecular subtypes and genetic alterations for evaluating clinical outcomes in 1029 patients with endometrial cancer. Br. J. Cancer.

[26] Stelloo E., Jansen A.M.L., Osse E.M., Nout R.A., Creutzberg C.L., Ruano D., Church D.N., Morreau H., Smit V., van Wezel T. (2017). Practical guidance for mismatch repair-deficiency testing in endometrial cancer. Ann. Oncol..

[27] Scheiderer A., Riedinger C., Kimball K., Kilgore L., Orucevic A. (2022). Reporting Subclonal Immunohistochemical Staining of Mismatch Repair Proteins in Endometrial Carcinoma in the Times of Ever-Changing Guidelines. Arch. Pathol. Lab. Med..

[28] Dillon J.L., Gonzalez J.L., DeMars L., Bloch K.J., Tafe L.J. (2017). Universal screening for Lynch syndrome in endometrial cancers: Frequency of germline mutations and identification of patients with Lynch-like syndrome. Hum. Pathol..

[29] Watkins J.C., Nucci M.R., Ritterhouse L.L., Howitt B.E., Sholl L.M. (2016). Unusual Mismatch Repair Immunohistochemical Patterns in Endometrial Carcinoma. Am. J. Surg. Pathol..

[30] Markow M., Chen W., Frankel W.L. (2017). Immunohistochemical Pitfalls: Common Mistakes in the Evaluation of Lynch Syndrome. Surg. Pathol. Clin..

[31] Loughrey M.B., Dunne P.D., Coleman H.G., McQuaid S., James J.A. (2019). Punctate MLH1 mismatch repair immunostaining in colorectal cancer. Histopathology.

[32] Kommoss S., McConechy M.K., Kommoss F., Leung S., Bunz A., Magrill J., Britton H., Kommoss F., Grevenkamp F., Karnezis A. (2018). Final validation of the ProMisE molecular classifier for endometrial carcinoma in a large population-based case series. Ann. Oncol..

[33] Stelloo E., Nout R.A., Osse E.M., Jurgenliemk-Schulz I.J., Jobsen J.J., Lutgens L.C., van der Steen-Banasik E.M., Nijman H.W., Putter H., Bosse T. (2016). Improved Risk Assessment by Integrating Molecular and Clinicopathological Factors in Early-stage Endometrial Cancer-Combined Analysis of the PORTEC Cohorts. Clin. Cancer Res..

[34] Talhouk A., McConechy M.K., Leung S., Li-Chang H.H., Kwon J.S., Melnyk N., Yang W., Senz J., Boyd N., Karnezis A.N. (2015). A clinically applicable molecular-based classification for endometrial cancers. Br. J. Cancer.

[35] Talhouk A., McConechy M.K., Leung S., Yang W., Lum A., Senz J., Boyd N., Pike J., Anglesio M., Kwon J.S. (2017). Confirmation of ProMisE: A simple, genomics-based clinical classifier for endometrial cancer. Cancer.

[36] Committee on Practice Bulletins-Gynecology, Society of Gynecologic Oncology (2014). ACOG Practice Bulletin No. 147: Lynch syndrome. Obstet. Gynecol..

[37] Chen L., Han X. (2015). Anti-PD-1/PD-L1 therapy of human cancer: Past, present, and future. J. Clin. Investig..

[38] Yamaguchi H., Hsu J.M., Yang W.H., Hung M.C. (2022). Mechanisms regulating PD-L1 expression in cancers and associated opportunities for novel small-molecule therapeutics. Nat. Rev. Clin. Oncol..

[39] Chan T.A., Yarchoan M., Jaffee E., Swanton C., Quezada S.A., Stenzinger A., Peters S. (2019). Development of tumor mutation burden as an immunotherapy biomarker: Utility for the oncology clinic. Ann. Oncol..

[40] Le D.T., Durham J.N., Smith K.N., Wang H., Bartlett B.R., Aulakh L.K., Lu S., Kemberling H., Wilt C., Luber B.S. (2017). Mismatch repair deficiency predicts response of solid tumors to PD-1 blockade. Science.

[41] Mandal R., Samstein R.M., Lee K.W., Havel J.J., Wang H., Krishna C., Sabio E.Y., Makarov V., Kuo F., Blecua P. (2019). Genetic diversity of tumors with mismatch repair deficiency influences anti-PD-1 immunotherapy response. Science.

[42] Llosa N.J., Cruise M., Tam A., Wicks E.C., Hechenbleikner E.M., Taube J.M., Blosser R.L., Fan H., Wang H., Luber B.S. (2015). The vigorous immune microenvironment of microsatellite instable colon cancer is balanced by multiple counter-inhibitory checkpoints. Cancer Discov..

[43] Le D.T., Uram J.N., Wang H., Bartlett B.R., Kemberling H., Eyring A.D., Skora A.D., Luber B.S., Azad N.S., Laheru D. (2015). PD-1 Blockade in Tumors with Mismatch-Repair Deficiency. N. Engl. J. Med..

[44] O’Malley D.M., Bariani G.M., Cassier P.A., Marabelle A., Hansen A.R., De Jesus Acosta A., Miller W.H., Safra T., Italiano A., Mileshkin L. (2022). Pembrolizumab in Patients With Microsatellite Instability-High Advanced Endometrial Cancer: Results From the KEYNOTE-158 Study. J. Clin. Oncol..

